# The *Xylella fastidosa* RTX operons: evidence for the evolution of protein mosaics through novel genetic exchanges

**DOI:** 10.1186/s12864-018-4731-9

**Published:** 2018-05-04

**Authors:** Gregory A. Gambetta, Mark A. Matthews, Michael Syvanen

**Affiliations:** 1 Bordeaux Science Agro, Institut des Sciences de la Vigne et du Vin, Ecophysiologie et Génomique Fonctionnelle de la Vigne, UMR 1287, F– 33140 Villenave d’Ornon, France; 2 Department of Viticulture and Enology, University of California, Davis, CA 95616-8645 USA; 3 Department of Medical Microbiology and Immunology, School of Medicine, University of California, Davis, CA 95616-8645 USA

**Keywords:** Hemolysin, Pierce’s disease, Horizontal gene transfer, Lateral gene transfer, Orphan

## Abstract

**Background:**

*Xylella fastidiosa* (*Xf*) is a gram negative bacterium inhabiting the plant vascular system. In most species this bacterium lives as a benign symbiote, but in several agriculturally important plants (e.g. coffee, citrus, grapevine) *Xf* is pathogenic. *Xf* has four loci encoding homologues to hemolysin RTX proteins, virulence factors involved in a wide range of plant pathogen interactions.

**Results:**

We show that all four genes are expressed during pathogenesis in grapevine. The sequences from these four genes have a complex repetitive structure. At the C-termini, sequence diversity between strains is what would be expected from orthologous genes. However, within strains there is no N-terminal homology, indicating these loci encode RTXs of different functions and/or specificities. More striking is that many of the orthologous loci between strains share this extreme variation at the N-termini. Thus these RTX orthologues are most easily visualized as fusions between the orthologous C–termini and different N-termini. Further, the four genes are found in operons having a peculiar structure with an extensively duplicated module encoding a small protein with homology to the N-terminal region of the full length RTX. Surprisingly, some of these small peptides are most similar not to their corresponding full length RTX, but to the N-termini of RTXs from other *Xf* strains, and even other remotely related species.

**Conclusions:**

These results demonstrate that these genes are expressed *in planta* during pathogenesis. Their structure suggests extensive evolutionary restructuring through horizontal gene transfers and heterologous recombination mechanisms. The sum of the evidence suggests these repetitive modules are a novel kind of mobile genetic element.

**Electronic supplementary material:**

The online version of this article (10.1186/s12864-018-4731-9) contains supplementary material, which is available to authorized users.

## Background

One of the goals of whole genome analysis of pathogenic bacteria is to identify novel genes encoding potential pathogenicity factors [1–3]. A reasonable approach is to compare the sequence of a pathogenic bacteria to a non-pathogenic relative [4] as illustrated by the complete sequencing and subsequent comparisons of several different strains of *Xylella fastidosa* (*Xf*) with different host-specificities, levels of virulence, etc. [5–9]. In general, this approach has run into the difficulty that two closely related strains from the same species can have differences in a large number of genes, many of which are unrelated to pathogenesis [5, 6]. In this work we have taken a different approach, comparing genes previously characterized as pathogenicity factors, focusing on those homologues with unusual divergence patterns across *Xf* strains.

*Xf* is an extremely fastidious gamma proteobacterium found only in the water conducting vascular system of diverse plant species. In most species *Xf* lives as a benign symbiote, but in several agriculturally important plants such as coffee, citrus, and grape, *Xf* is pathogenic [10]. In these susceptible plant species infection leads to leaf scorch, premature leaf senescence, reduced vigor, and crop loss. Mechanisms of its pathogenesis remain largely unknown. Originally, pathogenesis was thought to result from simple occlusion of the water conducting vascular system [11, 12], though other studies point towards a more nuanced mechanism where pathogenesis results from specific *Xf* pathogenicity factors and the induced plant defense response [13–15].

Based on comparative genome analysis our attention was drawn to a *Xf* gene family that encodes homologs of the *E. coli* hemolysins, members of the RTX protein family [16, 17]. RTXs are found in many bacterial human pathogens and are well established pathogenicity factors, appearing in surprisingly diverse disease processes. In animals, they are thought to disrupt host cell function leading to cytolysis through the formation of trans-membrane pores, but a variety of other functions have also been characterized in RTXs, including perturbing Ca^++^ signaling [18] and disruption of the cytoskeleton [19]. Such diverse functions should not be surprising given that in many cases RTXs actually represent protein mosaics, containing the conserved RTX domain that defines this protein family along with other functional domains of both known and unknown functions.

RTXs are also present in numerous plant pathogens and there is evidence for their role in plant disease [20] and in leaf lesion formation specifically [21]. In *Xf*, RTX-like adhesins have been implicated in pathogenesis [22–24]. Other RTXs have been hypothesized to play a role in *Xf* pathogenesis, although their expression has not been reported *in planta*. *Xf* 9a5c has been shown to produce and excrete two RTXs (RTX1 and RTX2 in this study) in culture [25], and whole genome comparisons of 9a5c with a nonpathogenic strain (J1a12) revealed that an RTX (RTX1) is among only 50 genes that are divergent in the nonpathogenic strain [8]. In addition, mutations in *Xf*’s type-I secretion system (via mutation of TolC), responsible for the secretion of RTXs, was shown to be essential for *Xf* survival *in planta* [26], although this interpretation is equivocal given the numerous proteins secreted via a TolC dependent pathway [27]. Taken together these results suggest a role for these loci in pathogenesis.

In this study we report gene expression analyses during Pierce’s disease (PD) in grapevine and comparative genomic analyses across *Xf* strains with specific host specificities; those causing Pierce’s disease in grapevine (Temecula), citrus variegated chlorosis (9a5c) [9, 28], almond leaf scorch (Dixon), and oleander leaf scorch (Ann1) [5, 6].

## Methods

### Operon sequences

The four putative RTX operon sequences analyzed from *Xf* strains causing Pierce’s disease in grapevine (Temecula), citrus variegated chlorosis (9a5c), almond leaf scorch (Dixon), and oleander leaf scorch (Ann1), were obtained through NCBI. The genomic sequence accession numbers for Temecula, 9a5c, Dixon, and Ann1, are AE009442, AE003849, AAAL02000001, AAAM03000044, respectively. The NCBI protein accession numbers and locus tags for each operon’s RTX can be found in Additional file 1: Table S1.

### *Xf* infected plant material

Field samples were obtained from a commercial vineyard of Chardonnay grapevines (Beringer Vineyards, Yountville, CA, USA). Early in the growing season (May–July) plants were identified that exhibited typical Pierce’s disease symptoms; shriveled fruit, leaf-scorch, petiole matchsticks, etc. Eight plants were selected and 10 leaves from each plant were harvested and homogenized. Total DNA was isolated and the presence of *Xf* bacteria was confirmed by PCR [13]. In August, from these plants 14 leaves exhibiting various severities of leaf scorch, and 4 leaves from healthy controls, were excised, immediately frozen in dry ice, transported from the field to the lab, and placed at − 80 °C.

### RNA preparation and qPCR analyses

Each leaf was homogenized under liquid nitrogen. Immediately, total RNA was extracted from 0.3 g of tissue following the procedure described in Iandolino et al. (2004) and treated with 0.5 U/μg RQ1 DNase (Promega). First strand cDNA was synthesized using 2 μg of RNA, 0.5 μM (dT)18 primer, and 50 U of M-MLV Reverse Transcriptase (Promega).

The following primer pairs were designed to amplify an approximately 100 bp fragment of each individual Temecula RTX gene: RTX1, forward 5′-gaaagactggttgactgccgagg-3′ and reverse 5′-gaccccggaaaccttgagcagcat-3′; RTX2, forward 5′-aacggccggaacatcctggttgga-3′ and reverse 5′-caggggcaacgatatgctctacgg-3′; RTX3, forward 5′-aatacgctcactcgattcgcc-3′ and reverse 5′-gcagcctgtcagaaattgtc-3′; RTX4, forward 5′-ggagatttgaatgagatacgc-3′ and reverse 5′-gggaaggattccgcaagtagc-3′. As a positive control, the primer pairs were first tested by amplifying the fragment from total *Xf* (Temecula) genomic DNA. Briefly, *Xf* (Temecula) was grown in culture and genomic DNA was isolated using a DNeasy Tissue Mini Kit (Qiagen, Inc.) following the manufacturer’s protocol. Fragments were amplified in a 20 μL reaction volume including 1× buffer, 1.5 mM MgCl, 200 μM dNTPs, 250 nM of each primer, 2.5 units Taq polymerase, and approximately 100 ng *Xf* genomic DNA. The cycling conditions were as follows: 95 °C for 10 min, 30 cycles of 95 °C for 30 s, 60 °C for 30 s, and 72 °C for 1 min, followed by 72 °C for 5 min.

Quantitative real-time PCR was carried out in an ABI PRISM 7500 fast sequence detector (Applied Biosystems). Standard curves were produced for each RTX gene by diluting known concentrations of *Xf* to obtain a series at 1 log_10_ intervals as described in Gambetta et al., 2007 [13]. Each reaction (20 μL) contained 250 nM of each primer, 1 μL of template DNA, and 10 μL of Power SYBR Green Master Mix (Applied Biosystems). Thermal cycling conditions were as follows; 95 °C for 10 min, 40 cycles of 95 °C for 10 s and 60 °C for 1 min. Each cDNA sample was run in triplicate. Expression from symptomatic leaves was detected similarly, but with 5 μL of 1:10 diluted cDNA as template. PCR products were gel purified using a Qiaquick Gel Extraction Kit (Qiagen, Inc.) following the manufacturer’s protocol and then confirmed by direct sequencing. Primer only (for false positives via primer dimer) and non-reverse transcribed RNA (for false positives via residual DNA contamination) negative controls were included for all analyses.

### Phylogenetic and comparative analyses

Multisequence alignments were performed using Clustal [29] and the aligned sequences were edited with the sequence editor G blocks [30]. These sequences were then submitted to phylogenetic analysis using Phylip ver3.6 (distributed by the author, Felsenstein, J., Department of Genome Sciences, University of Washington, Seattle) and distance trees using Protdist and Fitch were determined. Protein distances were calculated using the Jones, Thornton, Taylor distance matrix [31]. The dotplot analysis was performed using Dotter in the ms dos environment [32].

## Results

### Xf RTX expression in planta

**Fig. 1:**
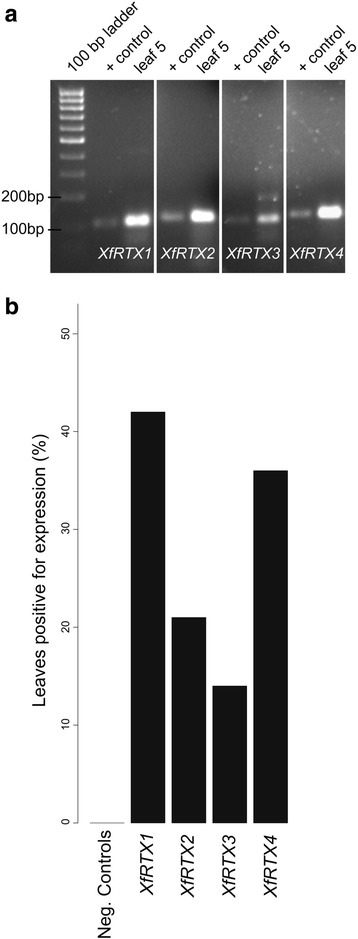
*In planta* expression of the *XfRTX* genes. **a** Agarose gel demonstrating *Xf* RTX qPCR products. (+ controls), amplified from *Xf* (Temecula) genomic DNA, and (leaf 5), qPCR product amplified from one of the 14 leaves tested for each RTX primer pair. All qPCR products were gel purified and sequenced to confirm identity. **b** Percentage of leaves that tested positive for *XfRTX* expression. Negative controls were leaves taken from asymptomatic plants

Our attention was drawn to the *RTX* genes from *Xylella fastidosa* after simple sequence examination showed that these potential pathogenicity factors appeared as a four locus gene family in all three strains but that there were unusual patterns of diversification (see below). Therefore, we set out to see if these four loci were expressed in *Xf* infected plants. Utilizing qPCR we demonstrated that all four Temecula *RTX*s are expressed during pathogenesis in naturally infected field grown Chardonnay grapevines. Primer pairs were developed to each Temecula *RTX* and were validated by conventional PCR (Fig. 1, positive controls). Further, we constructed qPCR standard curves (data not shown), prepared cDNA from symptomatic leaves, and assessed *RTX* expression in each leaf. We detected expression of *RTX1*, *RTX2*, *RTX3*, and *RTX4*, in 42, 21, 14, and 36% of the leaves tested, respectively (Fig. 1b). Leaves from asymptomatic controls showed no product. Products were gel isolated and sequenced to confirm their identities and proper negative controls were included to discount the possibility of false positives (see Materials and Methods).

### *Xf* RTX phylogeny

**Fig. 2:**
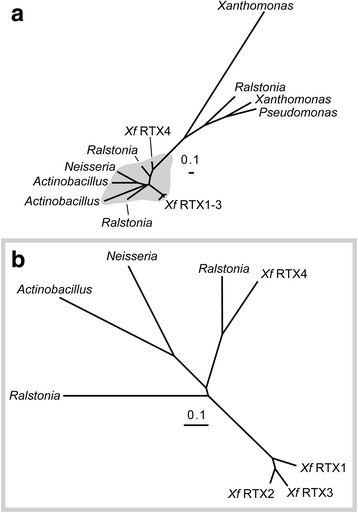
**a**, **b** Unrooted dendograms demonstrating the relationship between the conserved regions (coding regions excluding the hypervariable N-termini) *Xf* and other related organisms’ RTX peptide sequences. The Xf RTXs and the genus of the other organisms are noted. **b** Magnification of the shaded portion of the tree in **a**. Trees were created using the Phylip ver3.6 and distance scale is shown

**Fig. 3:**
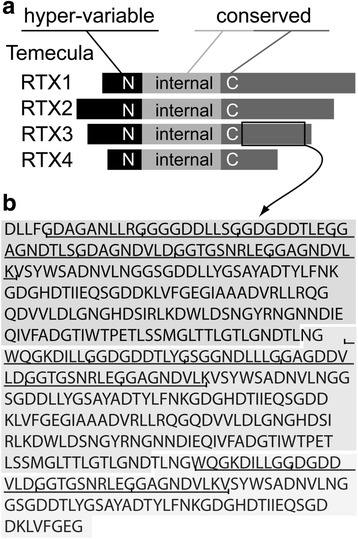
*Xf* (Temecula) RTX proteins. **a** Schematic of the Temecula RTX proteins delimited into the hyper-variable N-terminus (N), internal sequences (internal), and C-terminus (C). For a comprehensive comparison of Temecula and 9a5c N- and C-terminal sequence identities see Table 1. **b** Amino acid sequence of the C-terminal region of the Temecula RTX3 protein containing the characteristic nonapeptide repeats (underlined)

*Xf* contains four RTX loci [28]. The gene family is relatively diverse with the homologous portions of the genes showing much greater similarity between orthologues from different strains than seen between the different paralogous loci (Fig. 2). Phylogenetic analysis of the conserved domains of the *Xf* RTXs (i.e. the coding regions excluding the hypervariable N-termini; Fig. 3) display an evolutionary pattern expected from population isolation and vertical evolution from a common *Xf* ancestor that also carried the four RTX orthologues (Fig. 2). RTX1, RTX2, and RTX3 are more closely related to each other than they are to RTX loci from other related organisms. RTX4 is more closely related to the bacteriocin RTX-like protein first described in *Rhizobium leguminosarum* [33]. On the other hand, the *Xf* RTXs are highly diverged from RTXs from closely related *Xanthamonas* and *Pseudomonas* species. In general *Xf* RTXs are much more similar to those found in *Ralstonia*, *Neisseria*, and *Actinobacillus* species.

### Variation in the N-terminal region of *Xf* RTXs

The four *Xf* RTXs contain different N-termini, in contrast to the remaining, highly conserved regions of the protein including the C-terminal RTX domain (Fig. 3). The hyper-variable N-termini are of variable lengths and extend from the protein start to a conserved DPL(V/A)LDLD motif. The C-terminal RTX domain is the region containing the characteristic nonapeptide repeats (Fig. 3). Within strains there is no detectable N-terminal sequence similarity. For example, N-terminal sequence identities between the four RTX paralogues within *Xf* Temecula range from 7 to 17%, while C-terminal identities are what would be expected from orthologous genes ranging from 30 to 93% even including the more distant RTX4 (Fig. 3b and Table 1). RTX1 and RTX4 exhibit N-terminal hyper-variability between orthologues of Temecula and 9a5c (12–26% identity), while RTX2 and RTX3 have highly conserved N-termini (91% identity). These differences in N-terminal homology between strains are coincident with structural differences in the operons as a whole (Additional file 1: Figures S1 and S2).

**Table 1:** Sequence identities between the N-terminal and C-terminal regions of the *Xf* Temecula and 9a5c RTXs

		Temecula							
		N-terminal^*^				C-terminal^*^			
		RTX1	RTX2	RTX3	RTX4	RTX1	RTX2	RTX3	RTX4
Temecula	RTX1	100				100			
	RTX2	10	100			75	100		
	RTX3	13	17	100		93	72	100	
	RTX4	13	16	7	100	35	31	30	100
9a5c	RTX1	12	10	10	11	91	72	85	32
	RTX2	10	91	17	16	92	72	87	29
	RTX3	16	18	91	7	85	74	86	29
	RTX4	14	16	17	26	32	28	32	90

*Sequence relatedness of N-terminal (from the start to DPL(V/A)LDLD) and C-terminal (from the first GGX motif to stop) portions of *Xf* RTXs. (Blosum Matrix, Gap open-1, end gap-10, gap extension 1, gap distance-10)

### Operon structure

**Fig. 4:**
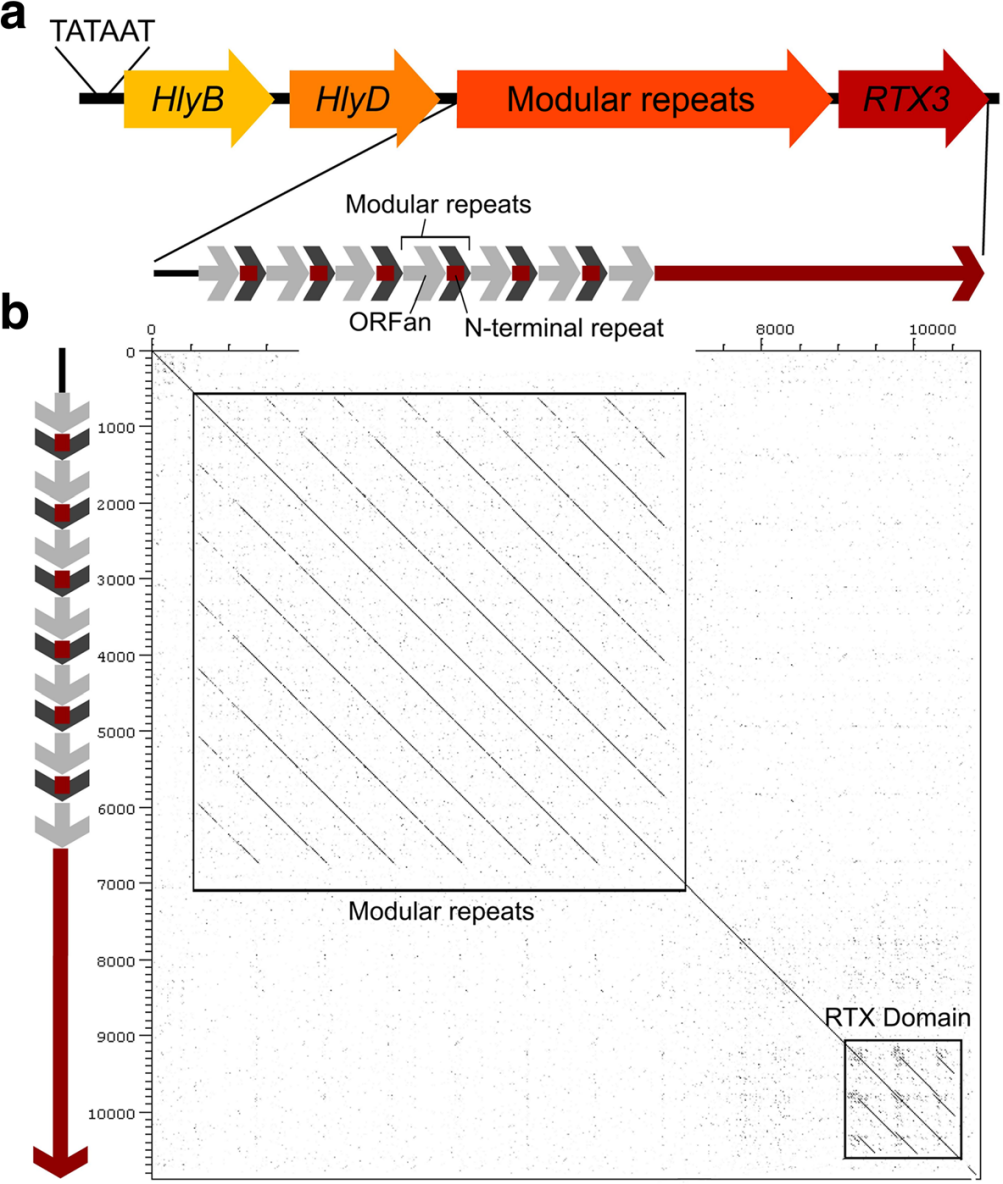
Structure of the *Xf* (Temecula) RTX3 operon. **a** The RTX3 coding region is preceded by the modular repeats, HlyB and HlyD genes, and the Pribnow-Schaller box (TATAAT). **b** DNA-DNA dot plot (created with the Dotter software) representing a self-comparison of the modular repeats and RTX3 coding region. The Temecula RTX3 operon contains six modular repeats including the N-terminal repeats (red) and ORFans (grey); arrows of the same color are homologous

Through similar analyses we compared orthologous operons across all four strains (Additional file 1: Figures S1 and S2). In general, all four RTX operons share a common structure: a region of tandem duplications of a module containing various numbers of putative ORFs (referred to as a “modular repeat”), followed by the full length RTX itself (e.g. Fig. 4a, Additional file 1: Figures S1 and S2). All ORFs contain putative − 35 sequences and Pribnow-Schaller boxes at their 5″ ends.

**Fig. 5:**
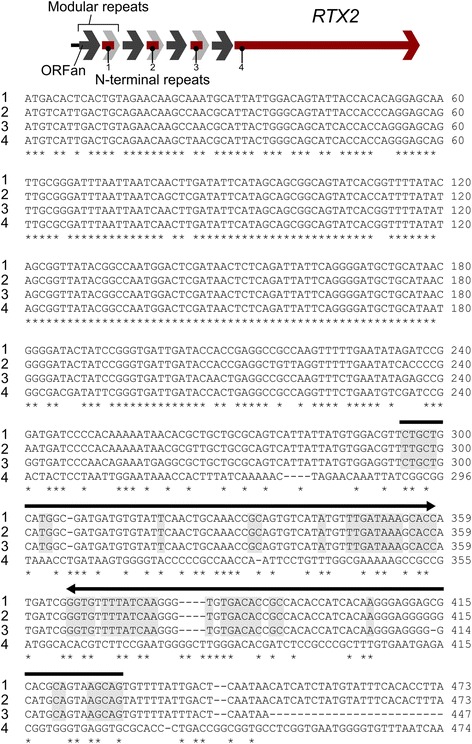
ClustalW multiple nucleotide sequence alignment of the *Xf* (Temecula) RTX2 operon N-terminal repeats (sequences 1–3) and the RTX2 N-terminus (sequence 4). N-terminal repeat sequences contain a large inverted repeat region (arrows and shading) that is coincident with a complete loss of homology with the RTX2 N-terminus

The RTX2 and RTX3 operons are similar across all strains, exhibiting regular tandem duplication of a modular repeat containing two putative ORFs lying upstream of the full length RTX (e.g. Figs. 4 and 5). One of the ORFs is nearly identical to the N-terminus of the full length RTX (the N-terminal repeats) while the other is an ORFan, in that it shares no homology with any protein in published databases. For example, a DNA-DNA dot plot comparing the Temecula RTX3 operon with itself illustrates the modular repeats in the 5′ portion of the operon (Fig. 4b). All strains differed in the number of modular repeats. The RTX1 and RTX4 operons have a more complex structure, with a short N-terminal repeat interspersed among clusters of ORFans. In some cases these clusters of ORFans are duplicated, in some cases rearranged, but the ORFs themselves remain intact and conserved.

Ann1 *RTX1* and Dixon *RTX3* both contain premature stop codons. Copies of the modular repeat in the Ann1 and Dixon RTX2 operons are frame shifted and contain a premature stop codon, respectively, and a modular repeat in the Ann1 RTX3 operon is frame shifted (Additional file 1: Figures S1 and S2).

### The modular repeats

The modular repeats in the RTX2 and RTX3 operons show little variation across strains. The individual modular repeats are highly conserved, and the loss of sequence similarity between the N-terminal repeat and the full length RTX is abrupt (Fig. 5). This conservation of sequences between the N-terminal repeat and the N-terminus of the corresponding RTX, followed by no observable similarity between the remaining module sequences and the remaining 3’end of the RTX gene is the classical signature of a gene fusion, or what is also referred to as a mosaic genetic pattern.

**Fig. 6:**
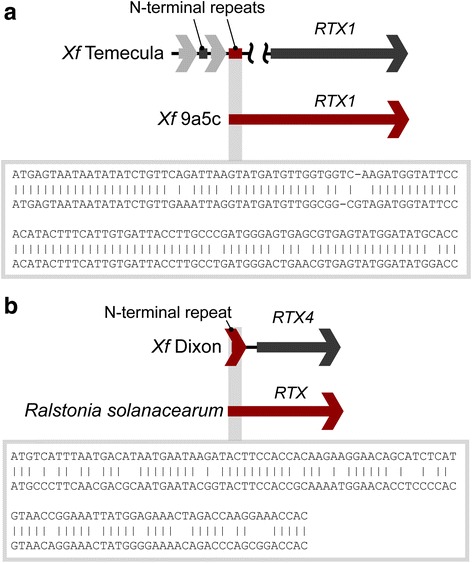
Nucleotide sequence alignments of N-terminal repeats with homology to other *Xf* strains and other organisms. **a** The *Xf* (Temecula) RTX1 operon contains an N-terminal repeat homologous to the N-terminus of RTX1 from strain 9a5c. **b** The *Xf* (Dixon) RTX4 operon contains an N-terminal repeat homologous to the N-terminus of RTX4 from *Ralstonia solanacearum*

In the RTX1 and RTX4 the pattern of the modular repeats are more complicated and there is more variation between modules. Some modules resemble the RTX N-terminus-ORFan pattern seen in the RTX2 and RTX3 operons while others are either shorter or even completely different. Surprisingly, some of the N-terminal repeats are most similar not to their corresponding full length RTX, but to the N-termini of RTXs from other *Xf* strains, and even other organisms (Fig. 6). For example, the Temecula RTX1 operon contains two short N-terminal repeat fragments, one sharing homology to its corresponding RTX, and another nearly identical to the non-homologous N-terminus of 9a5c RTX1 (Fig. 6a). The Ann1 and Dixon RTX4 operons contain an N-terminal repeat whose closest relative is the N-terminus of a RTX from *Ralstonia solanacearum* (Fig. 6b), a bacterium only remotely related to *Xf*.

### Inverted repeats, Rhs-like sequences, and GC content of the modules

The modular repeats of the RTX2 and RTX3 operons contain inverted repeat sequences. In the RTX2 operon an inverted repeat spans a large portion of the 3′ end of the ORFan (Fig. 5). The RTX3 modular repeat also has a smaller inverted repeat sequence (Additional file 1: Figure S3). In both case the repeat structures lie close to the homology discontinuity when the modular repeats are aligned with the adjacent full length RTX (Fig. 5 and Additional file 1: Figure S3). Such inverted repeats are often associated with special recombination or transposition activities that may very well be a part of the underlying mechanism that is producing the modular and mosaic patterns we are describing here.

**Fig. 7:**
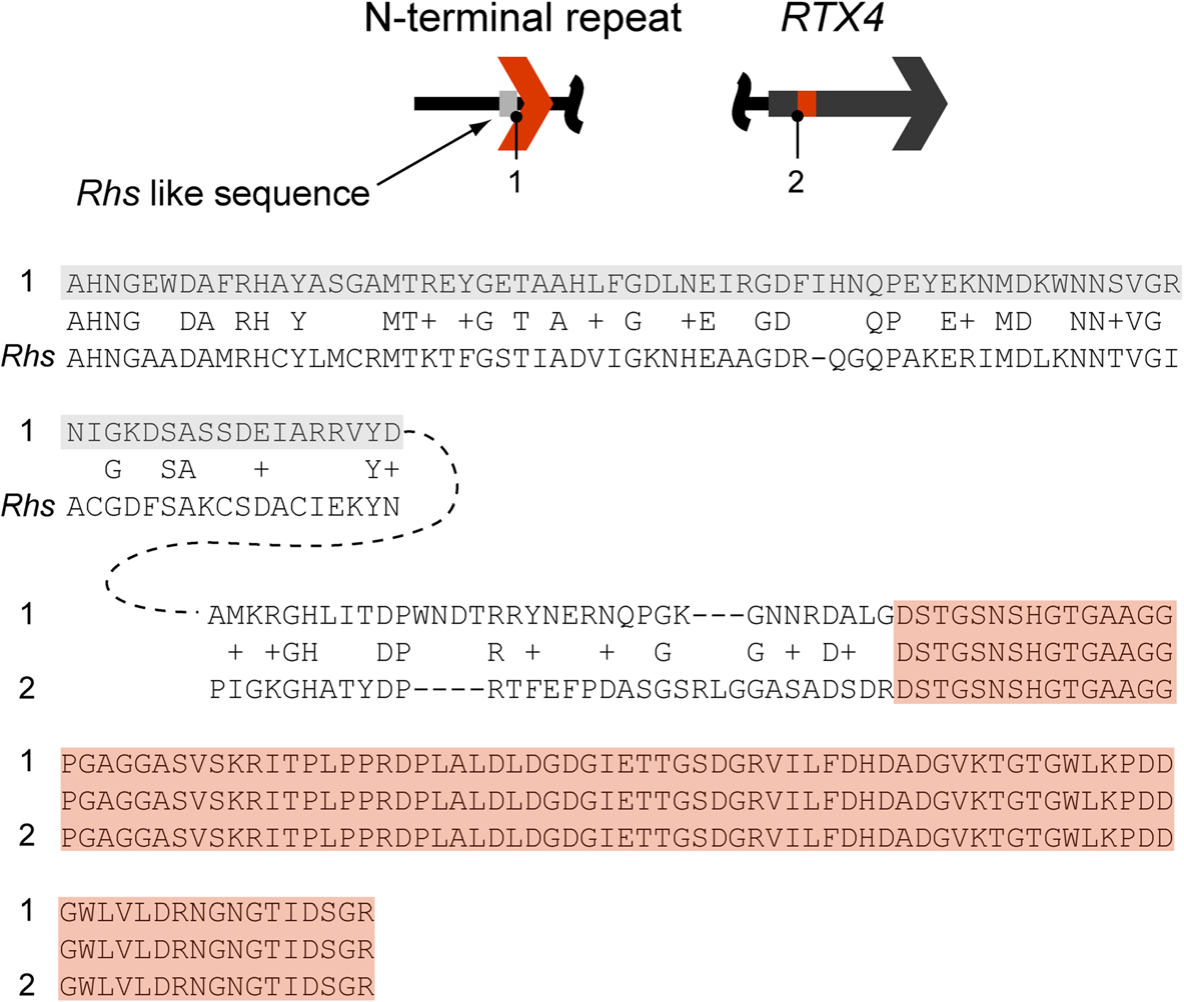
The *Xf* (Temecula) RTX4 operon N-terminal repeat (sequence 1) is a chimera between sequences with homology to Rhs sequences (grey shading) and the RTX4 N-terminus (orange shading). The aligned Rhs sequence is from *Escherichia coli* (NCBI Accession YP_001461405). Alignments are split but the N-terminal repeat sequence is continuous (dotted line)

The modular repeats within the RTX1 and RTX4 operons were different. For example, the Temecula RTX4 operon contains a small N-terminal repeat, unique to this *Xf* strain, that shares homology to the *E. coli* Rhs protein family (E values of approximately 10^− 5^)(Fig. 7). This ORF also exhibits a mosaic genetic pattern, with an abrupt transition from sequences homologous to Rhs to those homologous to the full length RTX.

The modular repeats at the 5’ends of the four RTX operons display many patterns expected of mobile genetic elements (e.g. involvement in mosaic patterns and linkage to sequences that appear to be involved in heterologous recombination mechanisms). In addition, GC content of these modular repeats have low GC content (41–44%) when compared to the rest of the *Xf* genome (52.7%, [28]) or even to the rest of the RTX operon (54%). Reduced GC content like this is a signature of DNA involved in horizontal gene transfer [34].

## Discussion

### Xf RTX expression in planta

To date there has been no direct evidence implicating the *Xf* RTXs in pathogenesis. In this study we demonstrated that all four genes are expressed during pathogenesis in grapevine. However, *Xf RTX* expression was not detected in all the leaves tested possibly due to the patchy distribution of *Xf in planta* [13] or alternatively because they may be expressed only under particular conditions, or during specific developmental time frames. We did not quantify expression of the *XfRTXs* in culture and thus they may be constitutively expressed.

In general, the approach utilized in this study suffers from the limitation that total mRNA, derived from both the plant and *Xf*, is being isolated. Thus, *Xf* concentrations and/or *Xf RTX* expression must be great enough to overcome dilution resulting from plant derived message. It is for this reason that despite utilizing a qPCR based detection method we did not present quantitative data in this study. Future quantification of gene expression and its relation to *Xf* concentrations *in planta* should utilize an existing immunocapture or related technique in order to enrich for *Xf* derived message.

### Genetic exchange and operon structure

The direct modular repeats at the 5′ end of the RTX operon have many of the hallmarks of mobile genetic elements: (1) The inverted repeated sequences flanking the module in RTX2 and 3 and the appearance of an *rhs* sequence at a modular junction in RTX4 are features that are associated with known mobile genetic elements from insertion sequences to pathogenicity islands. (2) The GC content of the modules averages approximately 44%, significantly lower than the GC content of the *Xf* genome. Regions of lowered GC content are a near universal signal of mobile genetic elements from those as small as insertion sequences to large pathogenicity islands, reflecting the heterologous DNA recombination events during their histories [34]. (3) One of the modules in RTX4 has its closest relative in *Ralstonia*, though the sequence is completely absent in two of the *Xf* strains as well as *Xf*’s closer relatives. And, (4) the RTX genes appear as mosaics.

### *Xf* RTXs; protein mosaics

The *Xf* RTXs are protein mosaics with their hyper-variable N-termini creating non-homologues at the N-terminus and true orthologues at the C-terminus. This is consistent with the modular nature of these proteins along with their distinct functional domains that have been noted before. For example the structure of the *Legionella pneumophila* RTX gene suggests recombination has resulted in tandem duplications of some domains; reminiscent of the *Xf* RTX operon structure characterized in this study [35], but this is the first example where the hypervariable N-terminal portion of the protein is included in the repeat. The precise mechanisms that bring about the mosaic structure in many RTX proteins are unknown, but in some cases there is clear evidence that the genes encoding these proteins have evolved through horizontal gene transfer from both within and between species (discussed below). The large diversity of biochemical functions attributed to RTXs is likely related to the mosaic structure of RTX proteins. To what extent this uncharacterized N-terminal protein domain, and the genetic exchanges therein, contribute to *Xf* pathogenesis or host specificity is unknown, but worthy of future investigation.

### RTX genes, ORFans, and horizontal gene transfer

In general the *Xf* RTX loci and their individual modules show evidence of being involved horizontal gene transfer events. The majority of *Xf*’s closer relatives, the gamma proteobacterum *Xanthamonas* and *Pseudomonas* species do not contain RTXs, and those that do contain highly divergent proteins. The most closely related *Xanthamonas* RTX, from *Xanthamonas axonopodis*, also appears to have been acquired horizontally [36], and there are numerous other cases in which the acquisition of RTXs appears to have occurred through horizontal gene transfer; the *Mannheimia* (*Pasteurella*) *haemolytica* leukotoxins [37–39], a thermostable hemolysin among *Vibrio* species [40], and the *Vibrio cholera* RTX [41], among others. Furthermore, like the *Xf* RTXs characterized here, RTX coding regions in some other organisms are associated with mobile genetic elements such as *Rhs*/*vgr* sequences [41, 42] and plasmids [43].

ORFans (called “orphans” when described in Eukarkotic genomics literature) are open reading frames that have been found in whole genome sequences that have no identifiable homologs in other, even very closely related, taxonomic groups. Given that there is no sequence homology within the protein database’s these are almost by definition of unknown function. These ORFs are not simple sequencing artifacts but represent real genes that are expressed and contribute to organismal phenotype [44]. At this point the simplest explanation is that they are recently evolved genes, at least since the last bifurcation from the given taxa’s last common ancestor. *Xf* RTX4 in our study of *Xf* looks like an ORFan but one homolog is found in a distantly related *Ralstonia* strain suggesting a horizontal gene transfer of a recently evolved gene into another species that occupies a similar habitat. We may speculate that *Xf* RTX4 plays an important role in pathogenesis.

That the *Xf* RTXs appear more closely related to those from the beta proteobacterium *Ralstonia solanacearum* is noteworthy since *Ralstonia* is also a vascular plant pathogen. Given the cytolytic effects of RTXs in other pathogenic bacteria a similar role in plant pathogenesis is plausible, especially considering these organisms’ niche environment; the non-living, nutrient poor, water conducting xylem. A cytolytic function, by which the pathogen manipulates it’s environment to make it more hospitable through obtaining nutrients from the surrounding, living, xylem parenchyma, would address the perplexing observation that they grow to such high densities in such a poor environment. Furthermore, it would reconcile the prevailing idea that pathogenesis results from vascular occlusion and water deficit with more contemporary studies questioning this hypothesis. As with cellular level water deficits, loss of membrane integrity leads to decreases in both water potential and turgor. The difference however is that loss of membrane integrity would lead to increases in solute potential (as opposed to decreases during cellular dehydration), which is what is observed during *Xf* pathogenesis [15, 45]. Here we provide evidence that genetic exchanges have occurred with *Ralstonia solanacearum* suggesting that their common niche environment may facilitate, and even favor, gene flow between the two organisms.

A variety of bacterial plant pathogens harbor genes encoding RTXs and this work provides the first steps investigating their role in pathogenesis. The uncanny similarities in structure and the high prevalence of genetic exchange (probably horizontally) in both plant and animal pathogen RTXs suggests they may play key roles in pathogenesis across kingdoms.

## Conclusions

RTXs are pathogenicity factors, widespread among gram-negative bacterial pathogens. To date, studies investigating the role of RTXs in pathogenesis have primarily focused on their effects in animal cells and tissues. This study provides the first direct evidence of their expression during pathogenesis in grapevine during Pierce’s disease. Through detailed sequence comparisons we show that the *Xf* RTX operons have a unique structure suggesting that the modular repeats that make up a large portion of these operons are novel mobile genetic elements.

## Additional file

Additional file 1: **Table S1.** Gene name, NCBI protein accession number, and NCBI gene locus tag for the Xylella fastidiosa RTXs. **Figure S1 and S2.** Complete DNA-DNA dot plots (created with the Dotter software) of RTX operon comparisons between *Xf* (Temecula) and itself, other *Xf* strains Dixon, 9a5c, and Ann1, and in the case of the RTX4 operon *Ralstonia solanacearum*. Putative open reading frames are designated by arrows, orthologues are like colored, and mutations resulting in premature stop codons or frame shifts (FS) are noted. **Figure S3.** ClustalW multiple nucleotide sequence alignment of the Xf (Temecula) RTX3 operon N-terminal repeats (sequences 1-6) and the RTX3 N-terminus (sequence 7). N-terminal repeat sequences contain a large inverted repeat region (arrows and shading). (PDF 809 kb)
