# Supplementary material for: The Xylella fastidosa RTX operons: evidence for the evolution of protein mosaics through novel genetic exchanges
Source: BMC Genomics. 2018 May 4;19:329. doi: 10.1186/s12864-018-4731-9 (PMC5935956; doi:10.1186/s12864-018-4731-9)
Supplement: Supplementary file 1 — Table S1. Gene name, NCBI protein accession number, and NCBI gene locus tag for the Xylella fastidiosa RTXs. Figure S1 and S2. Complete DNA-DNA dot plots (created with the Dotter software) of RTX operon comparisons between Xf (Temecula) and itself, other Xf strains Dixon, 9a5c, and Ann1, and in the case of the RTX4 operon Ralstonia solanacearum. Putative open reading frames are designated by arrows, orthologues are like colored, and mutations resulting in premature stop codons or frame shifts (FS) are noted. Figure S3. ClustalW multiple nucleotide sequence alignment of the Xf (Temecula) RTX3 operon N-terminal repeats (sequences 1-6) and the RTX3 N-terminus (sequence 7). N-terminal repeat sequences contain a large inverted repeat region (arrows and shading). (PDF 809 kb) [file 12864_2018_4731_MOESM1_ESM.pdf]

Supplemental Tables and Figures

Supplemental Table 1. Gene name, NCBI protein accession number, and NCBI gene locus tag for the Xylella fastidiosa RTXs.

Temecula			
Gene name	Protein accession #	Locus tag	Peptide Length (AAs)
RTX1	NP_779700	PD1506	1605
RTX2	NP_778540	PD0305	1607
RTX3	NP_779625	PD1427	1406
RTX4	NP_780271	PD2097	999
9a5c			
RTX1	NP_297958	*XF*0668	1207
RTX2	NP_298301	*XF*1011	1636
RTX3	NP_299686	*XF*2407	2064
RTX4	NP_300036	*XF*2759	1296
Dixon			
RTX1	ZP_00650795	*Xf*asaDRAFT_1815	943
RTX2	ZP_00651923	*Xf*asaDRAFT_1327	1814
RTX3	ZP_00652468	*Xf*asaDRAFT_0690	435

RTX4	ZP_00652263	*Xf*asaDRAFT_0865	1642
Ann1			
RTX1	ZP_00682471	*Xf*asoDRAFT_2218	943
RTX2	ZP_00680044	*Xf*asoDRAFT_4106	1814
RTX3	ZP_00683274	*Xf*asoDRAFT_1707	1035
RTX4	ZP_00681241	*Xf*asoDRAFT_2517	1642

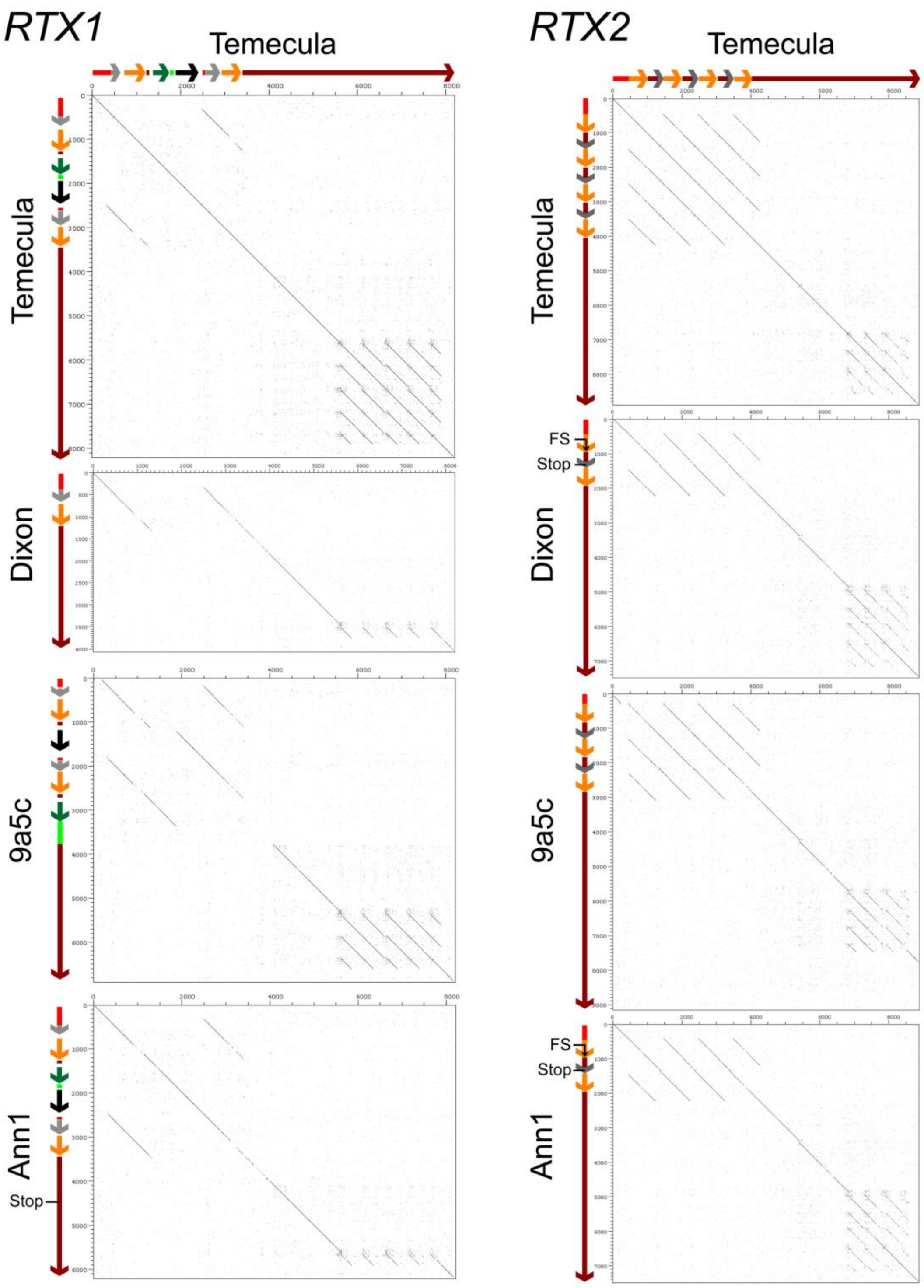

Supplemental Fig. S1. Complete DNA-DNA dot plots (created with the Dotter software) of RTX operon comparisons between *Xf* (Temecula) and itself, other *Xf* strains Dixon, 9a5c, and Ann1, and in the case of the RTX4 operon *Ralstonia solanacearum*. Putative open reading frames are designated by arrows, orthologues are like colored, and mutations resulting in premature stop codons or frame shifts (FS) are noted.

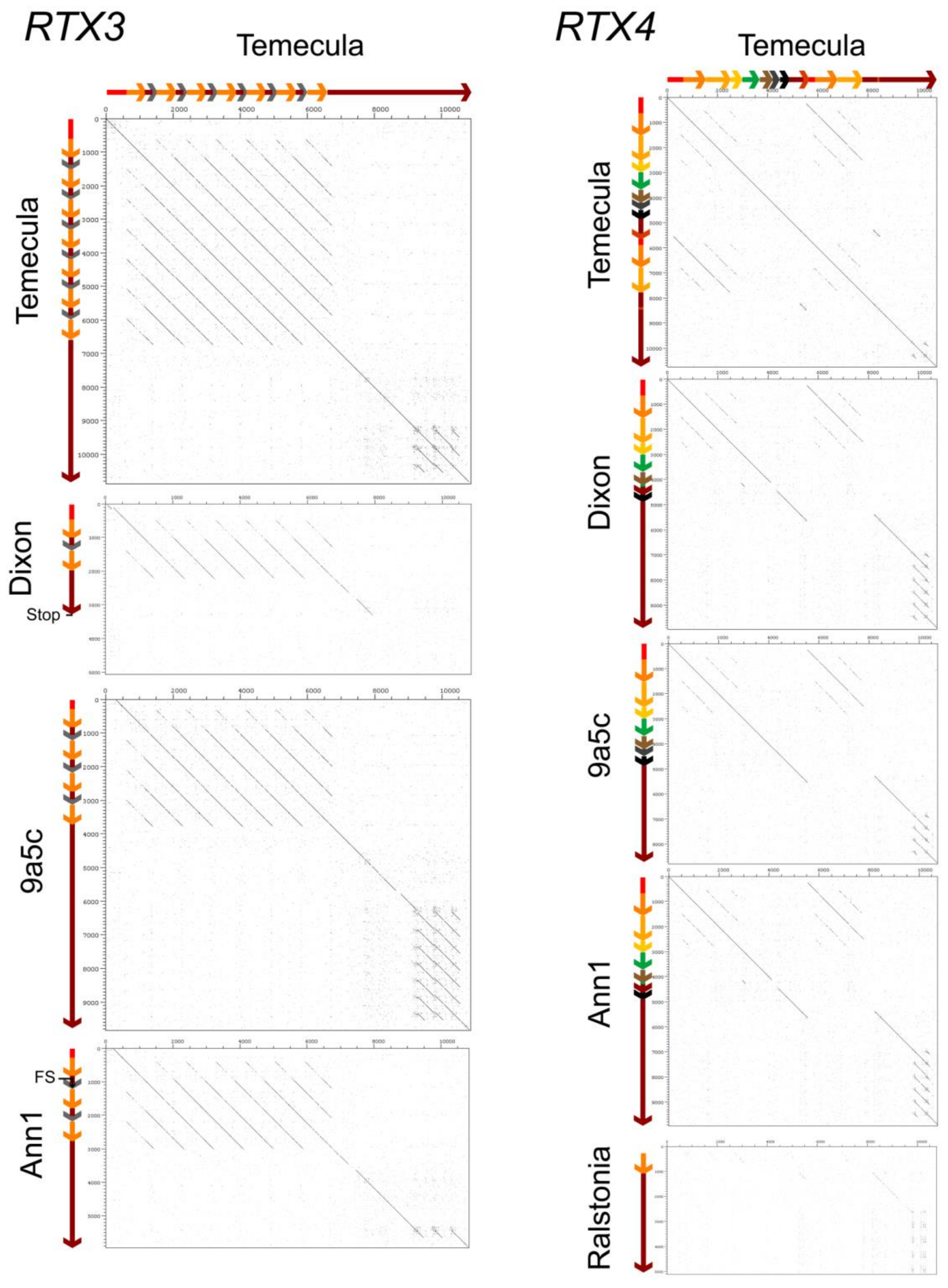

Supplemental Fig. S2. Complete DNA-DNA dot plots (created with the Dotter software) of RTX operon comparisons between *Xf* (Temecula) and itself, other *Xf* strains Dixon, 9a5c, and Ann1, and in the case of the RTX4 operon *Ralstonia solanacearum*. Putative open reading frames are designated by arrows, orthologues are like colored, and mutations resulting in premature stop codons or frame shifts (FS) are noted.

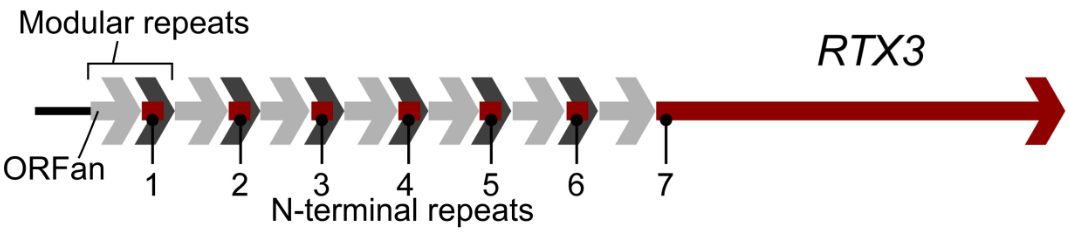


```
1 ATGATGACAAGTTTAGATTTTTATTTTGACCAAGCCACGTTGGACCGTGC 50
2 ATGATGACGAGTTTAGATTTTTATTTTGACCAAGCCACGTTGGACCGTGC 50
3 ATGATGACAAGTTTAGATTTTTATTTTGACCAAGCCACGTTGGACCGTGC 50
4 ATGATGACGAGTTTAGATTTTTATTTTGATCAAACAACGCTAAATCTTGC 50
5 ATGATGACAAGTTCATATTTTTATTTTGATCAAACAACGCTAGATCTTGC 50
6 ATGATGACAAGTTCAGATTTTTATTTTGATCAAGCCACGTTGGACCGTGC 50
7 ---ATGACGAATTTAGATTTTTATTTTGATCAGGCGACATTAGATCGTGC 47
     ***** * ** * ************* **  * **  *  * * ***

1 CAACACCTATAGAAATGCAGGGAATGACTGGGCGATGTACCACACATTAT 100
2 CAACACCTATAAAAATGCCGGGAATTATCCGGCGATGTACCAGACATTGT 100
3 CAACACCTATAGAAATGCCGGGAATGACTGCGCGATGTACCGGACATTAT 100
4 CAACACCTACAGCAATGCCGGGAATGACTGGGCGATGTACCACACATTAT 100
5 CAACACCTACAGCAATGCCGGGAATGACTGGGCGATGTACCAGACATTAT 100
6 CAACACCTATAAAAATGCCGGGAATTATCCGGCGATGTACCAGACATTGT 100
7 TGAGGCTTACAGAAATTCTGGGAATTATCCGGCGATGTACCAGACATTGT 97
    *  * ** *  *** * ****** *    **********  ***** *

1 CCGATGCCGTTAAAGCCGGTGGCGGCGATGCCAGATTGGCGAATGGGTTG 150
2 CCGATGCCGTCAGCGTCGGTGGCGGCGATGCCAGATTGGCGAATGGGTTG 150
3 CCGATGCCGTCAAAGCCAGTGGTGGCGATGCCAGATTGGCGAATGGGTTG 150
4 CCGATACCGTCAAAGCCAGTGGCGGCGATGCCAGATCGGCGAATGGGTTC 150
5 CCGATGCCGTTAAAGCCAGTGGCGGCGATGCCAGATTGGCGAATGGGTTC 150
6 CCGATGCCGTCAAAGCCAGTGGTGGCGATGCCAGATTGGCGAATGGGTTC 150
7 CCGATGCCGTCAGCGCCGGTGGCGGCGATACCAGATTGGTGAATTGGCTG 147
  ***** **** *  * * **** ****** ****** ** **** ** *

1 AAGAATGCGGGGGACATCCATTACGCAC------AAGACCGCGACGCACC 194
2 AAGAATGCGGGGGACATCTATTCCGCAC------AACGCCGCGACGCACC 194
3 AAGAATGCGGGGGACATCCATTCCGCAC------AACGCCGCGACGCACC 194
4 AAGAATGCGGGGGACATCCATTCCGCAC------AACGCCGCGACGCACC 194
5 AAGAATGCGGGGGACATCCATTCCGCAC------AACGCCGCGACGCACC 194
6 AAGAATGCGGGGGACATCCATTCCGGGC------AACACCGCGACGCACC 194
7 ACACTTGCCGGGCAGATTAATTCAGGGCGAGGGGAAAATTTCTACGCAGA 197
  *    *** *** * **  ***  *  *      **     * *****
```

Supplemental Fig. S3. ClustalW multiple nucleotide sequence alignment of the *Xf* (Temecula) RTX3 operon N-terminal repeats (sequences 1-6) and the RTX3 N-terminus (sequence 7). N-terminal repeat sequences contain a large inverted repeat region (arrows and shading).
